# The β-carboline Harmine Induces Actin Dynamic Remodeling and Abrogates the Malignant Phenotype in Tumorigenic Cells

**DOI:** 10.3390/cells9051168

**Published:** 2020-05-08

**Authors:** Ronan Le Moigne, Frédéric Subra, Manale Karam, Christian Auclair

**Affiliations:** 1 Centre National de la Recherche Scientifique, CNRS UMR 8113, Laboratoire de Biologie et Pharmacologie Appliquée, 94230 Cachan, France; ronan.lemoigne29@gmail.com (R.L.M.); fsubra@ens-paris-saclay.fr (F.S.); 2 AC Bioscience, Innovation Park, Ecole Polytechnique Fédérale de Lausanne, CH-1024 Ecublens, Switzerland; manale.karam@ac-bioscience.com; 3 Département de Biologie, École Normale Supérieure Paris-Saclay, Université Paris-Saclay, 94230 Cachan, France

**Keywords:** harmine, actin polymerization, cell adhesion, tumor reversion

## Abstract

Numerous studies have shown that alteration of actin remodeling plays a pivotal role in the regulation of morphologic and phenotypic changes leading to malignancy. In the present study, we searched for drugs that can regulate actin polymerization and reverse the malignant phenotype in cancer cells. We developed a cell-free high-throughput screening assay for the identification of compounds that induce the actin polymerization in vitro, by fluorescence anisotropy. Then, the potential of the hit compound to restore the actin cytoskeleton and reverse the malignant phenotype was checked in EWS-Fli1-transformed fibroblasts and in B16-F10 melanoma cells. A β-carboline extracted from *Peganum harmala* (i.e., harmine) is identified as a stimulator of actin polymerization through a mechanism independent of actin binding and requiring intracellular factors involved in a process that regulates actin kinetics. Treatment of malignant cells with non-cytotoxic concentrations of harmine induces the recovery of a non-malignant cell morphology accompanied by reorganization of the actin cytoskeleton, rescued cell–cell adhesion, inhibition of cell motility and loss of anchorage-independent growth. In conclusion, harmine induces the reversion of the malignant phenotype by a process involving the modulation of actin dynamics and is a potential anti-tumor agent acting principally through a non-cytotoxic process.

## 1. Introduction

The actin cytoskeleton is present in all eukaryotic cells [1], and together with its associated proteins play important structural and functional roles in cell morphology, cell adhesion, motility, exo- and endocytosis, as well as in cell division [2,3,4]. Growing evidence suggests that alteration of actin or actin remodeling plays a pivotal role in the regulation of morphologic and phenotypic changes leading to malignancy. Tumor cells often display a disorganized actin homeostasis [5,6,7], a consequence of the activation of numerous signaling pathways involving oncogene products such as Src [8], Abl [9] or small GTPases of the Ras super-family proteins (Rac, Rho and Cdc42) [7,10,11]. On the other hand, the inactivation of several actin-binding proteins, including tropomyosin, vinculin or α-actinin that have tumor suppressor functions, can result in the induction of transformation [12]. Furthermore, several of these actin-binding proteins are deregulated in many cancers. For example, gelsolin a protein involved in actin filament severing/capping, is under-expressed in 60–90% of human tumors including ovarian [13], breast [14], bladder [15], prostate [16] and lung [17] cancers. Therefore, restoring actin cytoskeleton homeostasis in cancer cells could be a potentially powerful strategy for cancer treatment.

Several approaches for targeting the actin cytoskeleton have been developed [18,19]. Although direct actin-targeting drugs, such as jasplakinolide and cytochalasin D, have shown significant growth inhibitory activity on cancer cells in vitro [20,21,22], neither has made it through to preclinical studies due to limited therapeutic window caused by pan-expression of actin in all cell composing human organism [23]. This has led to the development of agents that target downstream effectors of actin remodeling, such as Rac and Cdc42 GTPases, which are more specifically deregulated in cancer cells [24]. These actin regulatory protein-targeting drugs exhibited a selective inhibitory effect on tumor cell growth and have shown promising preclinical outcomes [25]. Thus, drugs that specifically target actin disorganization in cancer is a promising tool to reverse the tumor phenotype and counteract cancer growth and progression.

In NIH-3T3 cells expressing the EWS-Fli1 oncoprotein (E/F cells, for Ewing Fibroblast) developed in our laboratory [26], malignant transformation is accompanied by major actin cytoskeleton disruption leading to cell polarization and increased motility. In these transformed cells, zyxin, a protein that promotes actin nucleation and is implicated in the spatial control of actin filament assembly [27], was found to be under-expressed. The restoration of a normal level of zyxin expression in E/F cells induces the reversion of their transformed phenotype. Cells with the highest levels of zyxin recover a fibroblastic morphology, stress fibers, focal adhesions and cell–cell contacts; they exhibit reduced intrinsic motility, anchorage-independent growth and decreased tumor growth in nude mice [26]. Based on these observations, we suggest that the NIH-3T3 non-cancerous cells with their corresponding EWS-Fli1-transformed E/F cells represent a pertinent model to identify drugs that modulate actin dynamics and as a consequence would abrogate the transformed phenotype, in a manner comparable to zyxin.

In order to identify such molecules, firstly, we developed a cell-free high-throughput screening assay for compounds that increase the actin polymerization rate constant and the F-actin to G-actin ratio in the presence of cellular extracts [28]. Then, we assessed the potential of the hit compound to restore the actin cytoskeleton and reverse the malignant phenotype in the E/F cells, as compared to their non-transformed NIH-3T3 counterparts. This screening procedure allowed us to identify harmine as a stimulator of actin polymerization. Harmine is a β-carboline extracted from *Peganum harmala* [29] already known for its psychoactive effects [30] and for its cytotoxic activity in tumor cells at high concentrations [31]. We show herein that, at non-cytotoxic concentrations, harmine induces the reorganization of the actin cytoskeleton in malignant cells resulting in the recovery of cell–cell adhesion, a decrease in cell motility and the loss of the malignant character as indicated by the marked decrease in anchorage-independent cell growth. These effects referred to as tumorigenic phenotype reversion can be considered as a starting point for the development of a new strategy for the design of non-cytotoxic cancer therapeutics.

## 2. Materials and Methods

### 2.1. Chemicals

The library of natural products is from the French National Museum of Natural History (Sorbonne University). Harmine hydrochloride was purchased from Sigma (St. Louis, MO, USA) and jasplakinolide from Molecular probes (Eugene, OR, USA).

### 2.2. Cell Culture and Cell Transfection

The NIH-3T3 murine fibroblasts and B16-F10 murine melanoma cell lines were purchased from the ATCC. The EWS-FLI1-transformed NIH-3T3 (E/F) cells were obtained as previously described [26]. Briefly, NIH-3T3 fibroblasts were stably transduced by the cDNA encoding the type1 EWS-FLI1 fusion protein inserted downstream of the Mo-MuLV long terminal repeat in the pBabe-puro retroviral vector. These cells were grown in Dulbecco’s Modified Eagles’ Medium (DMEM) supplemented with 10% heat-inactivated newborn calf serum, 100 UI/mL penicillin and 100 µg/mL streptomycin (culture medium) (all from Gibco, ThermoFisher Scientific, Les Ulis, France) at 37 °C in a humidified 5% CO_2_ atmosphere. E/F cells were selected with 2.5 µg/mL puromycin (Sigma Aldrich, Merck, St. Quentin Fallavier, France).

### 2.3. Cell Lysate Preparation

Cells were trypsinized and washed twice at room temperature with wash buffer at pH 6.5 (135 mM NaCl, 2.7 mM KCl, 11.9 mM NaHCO_3_, 0.36 mM NaH_2_PO_4_, 2 mM MgCl_2_, 0.2 mM EGTA, 5.5 mM glucose and 0.3% BSA). Briefly, 5 × 10^7^ cells were suspended in a sonication buffer (10 mM Tris-HCl pH 7.5, 10 mM EGTA, 2 mM MgCl_2_) containing complete protease inhibitor mixture (Roche, Merck, St. Quentin Fallavier, France). Cells were lysed on ice by minimal sonication required to break the cells (5 s pulses on setting 4 of a Novodirect vibracell). The sonicated cells were centrifuged at 8000× *g* rpm (Sigma 4K15C centrifuge) for 30 min at 4 °C. The clear supernatant was carefully removed and filtered through a 0.45 µm filter. The protein concentration was determined using the Bradford method according to the manufacturer’s instructions (Bio-Rad, Hercules, CA, USA). The supernatant was supplemented with 150 mM sucrose, 0.2 mM ATP and 0.2 mM DTT were added for each mg/mL of total proteins. The cellular extracts were aliquoted, frozen in liquid nitrogen, and then stored at −80 °C. Extracts can be frozen at −80 °C for at least 3 months without loss of activity.

### 2.4. Alexa 488-Actin Mediated Steady-State Fluorescence Anisotropy Measurement Assay

All reactions were carried out at 22 °C and fluorescence anisotropy signal was recovered at 520 nm with excitation at 490 nm in a Beacon 2000 (Panvera, Madison, WI, USA). Alexa Fluor 488-coupled actin (actin-Alexa488, Molecular Probes, Eugene, OR, USA) was centrifuged at 35,000 rpm for 2 h at 4 °C to sediment residual actin polymers in a Beckman L5–50B ultracentrifuge. The ultracentrifuged actin concentration was calculated using the non-ultracentrifuged Alexa488-actin as a standard. The supernatant was aliquoted, frozen in liquid nitrogen and stored at −80 °C. Before each experiment, an aliquot of ultracentrifuged Alexa488-actin was diluted to a concentration of 1 mg/mL in G buffer (5 mM Tris pH 8.1, 2 mM CaCl_2_, 0.2 mM DTT, 0.2 mM ATP). Briefly, 3 µL of diluted Alexa488-actin was mixed in 167 µL G buffer and actin monomers anisotropy was measured before the addition of 4 µL of polymerization buffer (2.5 M KCl, 50 mM MgCl_2_, 25 mM ATP) and 20 µL of cellular extract at 2 mg/mL. Measurements were made each 10 s for 150–200 s. Actin monomers anisotropy value was subtracted, yielding the anisotropy enhancement (ΔmA). The data were fitted with the equation Y = Ymax. [1 − exp(−KX)]. The curves start at zero and ascend to Ymax that corresponds to the steady-state anisotropy value (ΔAU, Anisotropy Unit), with a rate constant K.

### 2.5. Purified Actin Polymerization Measurement Assay

Polymerization of purified actin was measured by changes in pyrene fluorescence using a fluorescence spectrophotometer (Safas Xenius, Safas SA, Monaco) as described previously [32]. Briefly, actin was purified from rabbit skeletal muscle acetone powder and monomeric Ca^2+^-ATP actin was purified by Sephacryl S-300 chromatography in G buffer (5 mM Tris-HCl, pH 8, 0.2 mM ATP, 0.1 mM CaCl_2_, 0.5 mM DTT, 0.1 mM azide). Actin was labeled on Cys-374 with pyrene iodoacetamide. Monomeric Mg^2+^-ATP-10%pyrene-actin is prepared by incubation of Ca^2+^-ATP-actin (9 µM black actin + 1 µM pyrene labeled actin) on ice with a 0.2 mM EGTA and an 11-fold MgCl_2_ concentration relatively to actin. Actin (2 µM) is polymerized by the addition of 1/10 KMEI 10× (500 mM KCl, 10 mM MgCl_2_, 10 mM EGTA and 100 mM HCl-imidazole, pH 7).

### 2.6. MTT Toxicity Test

Cells were seeded in quadruplicates into 96-well plates at a density of 4000 cells in culture medium containing either vehicle (untreated cells) or different concentrations of harmine (treated cells) and cultured at 37 °C, 5% CO_2_. After 72 h, cells were incubated with 0.5 mg/mL MTT (Sigma) for 2 h at 37 °C. Then, formazan crystals were dissolved in a buffer containing 10% SDS (*w*/*v*, Bio-Rad), 50% Dimethyl formamide (Sigma), pH 4.7. The absorbance of reduced formazan was measured at 580 nm.

### 2.7. Immunofluorescence

Cells were cultured on glass coverslips in 6-well plates (Falcon, BD, Bedford, MA, USA) at 80,000 cells/well in culture medium containing either vehicle (untreated cells) or harmine (treated cells). Immunofluorescence was carried out 72 h after plating (cells were ~50% confluent). Cells were fixed for 5 min in PBS containing 3% paraformaldehyde and permeabilized with 0.4% Triton X-100 (ThermoFisher Scientific, Les Ulis, France) in PBS for 5 min. Cells were then incubated for one hour with blocking buffer (3% bovine serum albumin in PBS) and then incubated for 20 min with the anti-zyxin antibody at 1:200 dilution (Synaptic System; Goettingen, Germany), N-cadherin and Beta-catenin antibodies at 1:1000 dilution (BD Pharmingen, Bedford, MA, USA) or incubated with phalloidin-FITC (265 mM, Sigma) for 20 min in blocking buffer. Cells treated with primary antibodies were then incubated with a 1:100 dilution of donkey anti-mouse IgG antibodies conjugated to TRITC (Jackson Immunoresearch, Baltimore, PA, USA) for 20 min at room temperature. Coverslips were mounted in Vectashield^™^ (Zymed, Carlsbad, CA, USA) and analyzed with a fluorescence microscope (Nikon, Melville, NY, USA).

### 2.8. Western Blot

Total proteins were extracted as described previously [26]. Proteins (20 µg) were boiled in a Laemmli buffer and electrophoresed on a 10% SDS-polyacrylamide gel using standard procedures. The proteins were electroblotted onto a nitrocellulose membrane in 20 mM sodium phosphate, pH 6.7. The membrane was blocked by 10% powder milk in TBST (20 mM Tris-HCl, pH 7.5, 500 mM NaCl, 0.1% Tween 20) for 1 h at room temperature. Incubation with the primary antibody anti-GAPDH (1/10,000 dilution, Abcam), anti-zyxin (1/2000 dilution), anti-N-cadherin (1/10,000 dilution) or anti-β-catenin (1/10,000 dilution) was carried out overnight at 4 °C in TBST. Incubation with a goat anti-mouse IgG secondary antibody, conjugated to HRP (1/10,000 dilution, Southern Biotech, Birmingham, AL, USA), was carried out for one hour at room temperature and revealed by enhanced chemiluminescence (West Pico -Pierce, ThermoFisher Scientific). The image bands were quantified using Image J (NIH, MD, USA).

### 2.9. Cell–Cell Aggregation Test

Cells were initially cultured in 25 cm^2^ flasks until confluency either in the absence or presence of various concentrations of harmine. The cells were further trypsinized in order to obtain a homogenous preparation of isolated cells. Isolated cells were then suspended in a standard culture medium and samples of 2 mL containing 2 × 10^6^ cells were incubated at 37 °C for 50 min with gentle rotating shaking (90 rpm). The occurrence of cell aggregates was assessed by microscopic observation using a phase-contrast microscope.

### 2.10. Measurement of Adhesion Forces between Cells

A micromanipulation technique was used to measure adhesion forces between cells as described previously [33]. Briefly, forces were measured on the stage of an inverted epifluorescence microscope (Leica) equipped with objectives of 63× (PL FLUOTAR; NA/0.7; C PLAN NA/0.75) and with a cooled CCD C5985 (Hamamatsu, Massy, France) or Coolpix 5000 camera (Nikon). Cells were manipulated at 37 °C with two micropipettes, each held by one micromanipulator connected to a combined hydraulic/pneumatic system and a pressure sensor making it possible to control and measure the aspiration applied to the cells. Micropipettes were pulled (model P-2000; Sutter Instrument, Novato, CA, USA), cut and fire-polished with a homemade microforge, such that their i.d. was 4.0–5.5 µm. The aspiration applied to the left pipette was measured using a pressure sensor (model DP103-38; Validyne, Northridge, CA, USA). Aspiration was monitored continuously during the separation process and the values recorded for each of the last two cycles in the series (*P*_n−1_ and *P*_n_) were used to calculate the separation force (SF) for each doublet using the equation: SF = π(*d*/2)^2^ (*P*_n−1_ × *P*_n_)/2, where *d* is the i.d. of the left pipette. Results for around 20 measurements were used to obtain the mean force of separation for a specific contact time.

### 2.11. Wound Migration Assay

Cells were trypsinized, seeded in 60-mm dishes in their culture medium containing either vehicle (untreated cells) or harmine (treated cells). Two days later, when the cells had reached confluency, a wound was made in the monolayer with a sharp, 1-mm thick plastic scraper. Twenty-four hours later, cells were examined by phase-contrast light microscopy.

### 2.12. Apoptosis Assay

Detection of apoptosis was performed using an Annexin V-FITC apoptosis detection kit I (BD Pharmingen, Torreyana, CA, USA) according to the manufacturer’s instructions. Briefly, cells were resuspended in binding buffer (10 mM HEPES/NaOH, pH 7.4, 140 mM NaCl, 2.5 mM CaCl_2_) and then incubated for 30 min at room temperature in the dark in the presence of propidium iodide (PI) and/or Annexin V-FITC prior to fluorescence-activated cell sorter (FACS) analysis.

### 2.13. Cell Cycle Analysis

Cells were trypsinized, washed twice with PBS and pelleted by centrifugation. The cells were then fixed in PBS containing 80% cold ethanol and stained with propidium iodide (PI) solution (20 mM EDTA, 250 µg/mL RNAse, 50 µg/mL PI) for 30 min in the dark then analyzed by flow cytometry (FACScalibur, BD Biosciences Franklin Lakes, NJ, USA).

### 2.14. Anchorage-Independent Growth Assay

Cells were resuspended at 500 cells in 2.5 mL culture medium supplemented with 1% methylcellulose (Sigma), plated in triplicate into uncoated 35-mm dishes and incubated at 37 °C in a humidified 5% CO_2_ atmosphere. After 3 weeks, macroscopic clones (diameter = >120 µm) were counted.

## 3. Results

### 3.1. Identification of Harmine as a Stimulator of Actin Polymerization Using a Steady-State Fluorescence Anisotropy Assay

In order to identify modulators of cellular actin dynamics, we have developed a cell-free screening assay based on the measurement of the incorporation of actin-Alexa488 molecules in polymerizing actin filaments by fluorescence anisotropy [28]. Briefly, a crude cellular extract is incubated in the presence of actin-Alexa488 used as tracer and the variation of Alexa488 fluorescence anisotropy, which depends upon the state of actin polymerization, is measured. Thus, the assay allows the relative level of polymerized actin to be determined under cellular conditions by the acquisition of the steady-state anisotropy value (ΔmA eq) corresponding to the reaction equilibrium, and the actin polymerization pseudo-first-order rate constant (k) in the cellular extract.

**Figure 1:**
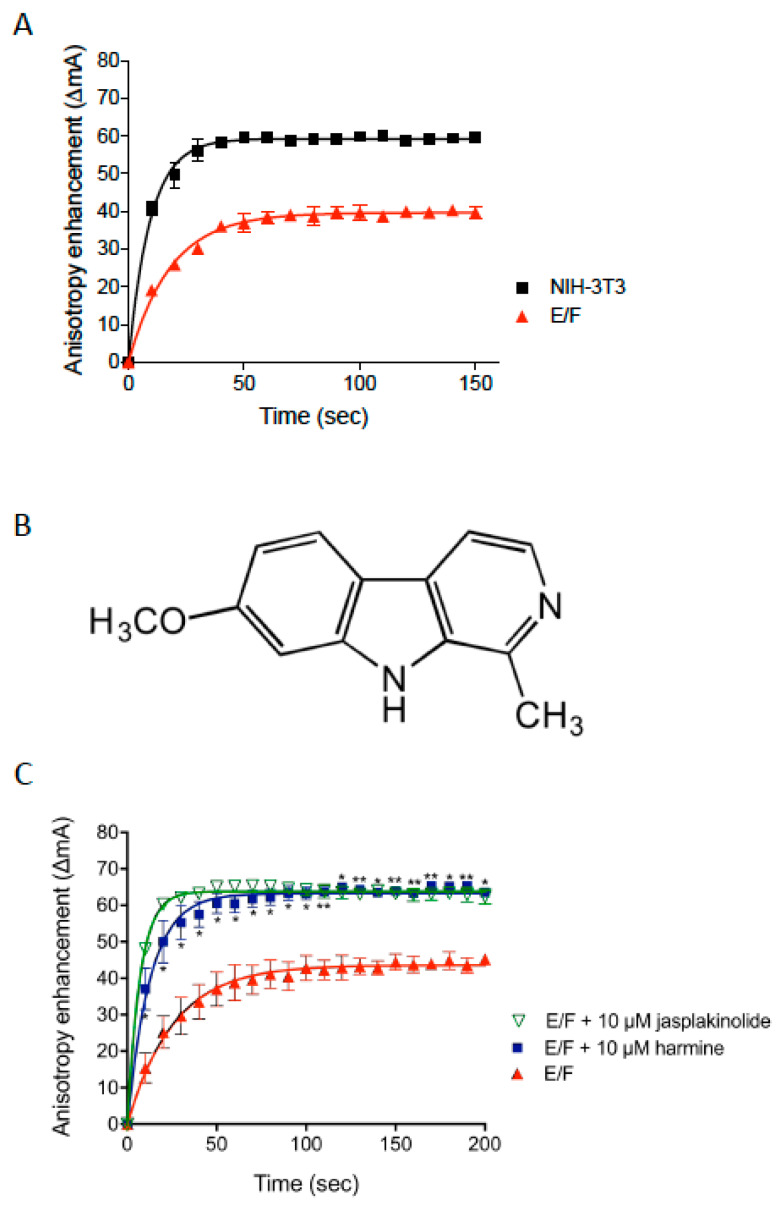
Effect of harmine on actin polymerization in EWS-FLI1 (E/F) cell extracts. (**A**) Kinetics of F-actin linear polymerization as measured in NIH-3T3 fibroblast and Ewing tumorigenic fibroblast (E/F) cell extracts using the fluorescence anisotropy assay. The results presented are means ± SEM for two independent experiments. (**B**) Chemical structure of harmine. (**C**) Kinetics of F-actin linear polymerization as measured in E/F cell extracts using the fluorescence anisotropy assay. Ten micromoles Jasplakinolide (green curve), 10 µM harmine (blue curve) or vehicle (red curve) are added, at time 0, to the polymerization buffer, actin-Alexa488 and E/F extracts. Actin polymerization was measured at different incubation times using fluorescence anisotropy assay. The results presented are means ± SEM for three independent experiments. * *p* < 0.05; ** *p* < 0.01 harmine-treated versus control untreated E/F cells.

The analysis of NIH-3T3 cellular extract by steady-state fluorescence anisotropy results in a ΔmA eq value of around 60 (*k* = 0.1) while the value obtained for the E/F cellular extract is decreased and yields a ΔmA eq of approximately 40 (*k* = 0.05, Figure 1A). These results demonstrate a clear difference in global actin dynamics between the non-tumorigenic NIH-3T3 fibroblasts and their tumorigenic counterpart E/F cells. The screening assay that permits the identification of modulators of actin dynamics is performed using E/F extracts and the result is considered as positive (hit) when the compound tested increases both the polymerization rate constant and the steady-state value.

A library of natural compounds was screened using this assay, and identified a methanolic extract of *Peganum harmala* seeds, that markedly increases both the actin polymerization rate constant and the F-actin steady-state value (Figure S1). Among the various alkaloids present in such extracts, harmine (Figure 1B), but not harmol and harmaline, displays the same properties as the *Peganum harmala* methanolic extract. Thus, in presence of harmine, the values of ΔmA eq and k of E/F extracts (ΔmA eq = 59; k = 0.08) significantly increased in comparison to untreated E/F extracts (ΔmA eq = 43; k = 0.038) and reached a level equivalent to that of control 3T3 cell extracts (Figure 1A,C). Consistently, the time to reach the equilibrium is higher in untreated E/F cells (T eq = 99.27 s) compared to harmine-treated E/F cells (T eq = 51 s, Figure 1C and Figure S2A). Similar observations were noted for half of the equilibrium values (Figure S2). This data shows that harmine enhances actin polymerization in E/F cell extracts. Almost identical results are obtained in the presence of jasplakinolide, a cyclic natural peptide is known to directly bind to F-actin resulting in the stabilization of actin in a filamentous form (Figure 1C and Figure S2). However, it should be emphasized that in the classical purified actin polymerization test (using pyrene-actin, 29), and in conditions where jasplakinolide increases the actin polymerization kinetics, harmine has no detectable effect (Figure S3). This strongly suggests that the effect of harmine on actin polymerization in cell extracts is not due to its direct binding to F-actin but rather action on other intracellular factors involved in a process that regulates actin kinetics.

### 3.2. Harmine Modifies EWS-Fli1-Transformed E/F Cell Morphology and Adhesion Proteins Localization

**Figure 2:**
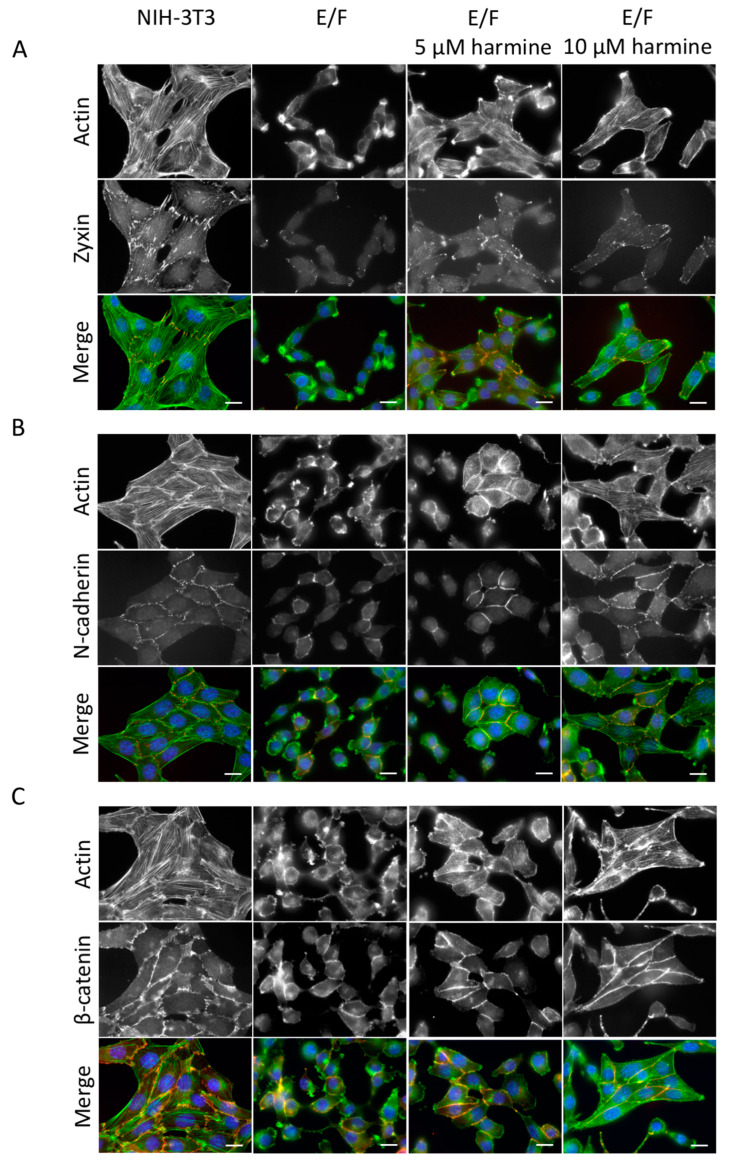
Effect of harmine on E/F cell morphology and cell–cell adhesion. NIH-3T3 and E/F cells were cultured in the absence or presence of 5 µM or 10 µM harmine, as indicated. After 72 h of culture, the cells were fixed, permeabilized and co-stained for actin with one of the following adhesion-related proteins; zyxin (**A**), N-cadherin (**B**) or β-catenin (**C**). Cell nuclei were stained with DAPI. Then, the cells were analyzed with a fluorescence microscope. In the merged images, actin is presented in green, zyxin, N-cadherin and β-catenin in red and nucleus in blue. Images are representative fields from three independent experiments. Scale bars: 10 µm.

We have shown previously that the transformation of NIH-3T3 cells by the expression of the oncogenic protein EWS-Fli-1 results in a marked cell shape modification accompanied by a spindle-like morphology and cell polarization with the occurrence of actin-rich lamellipodia and loss of cell–cell membrane adhesion [26]. Consistently, staining of F-actin (Figure 2A–C), zyxin (Figure 2A), N-cadherin (Figure 2B) and β-catenin (Figure 2C) shows numerous differences between NIH-3T3 and E/F cells. As shown in Figure 2 (first column), NIH-3T3 cells exhibit a typical fibroblast shape and a highly organized actin filament network, consisting of numerous, thick stress fibers connected to focal adhesions and intercellular contacts via well-defined zyxin organization (Figure 2A). Furthermore, N-cadherin (Figure 2B) and β-catenin (Figure 2C) staining show highly organized cell–cell contacts, characteristic of non-malignant cells.

**Figure 3:**
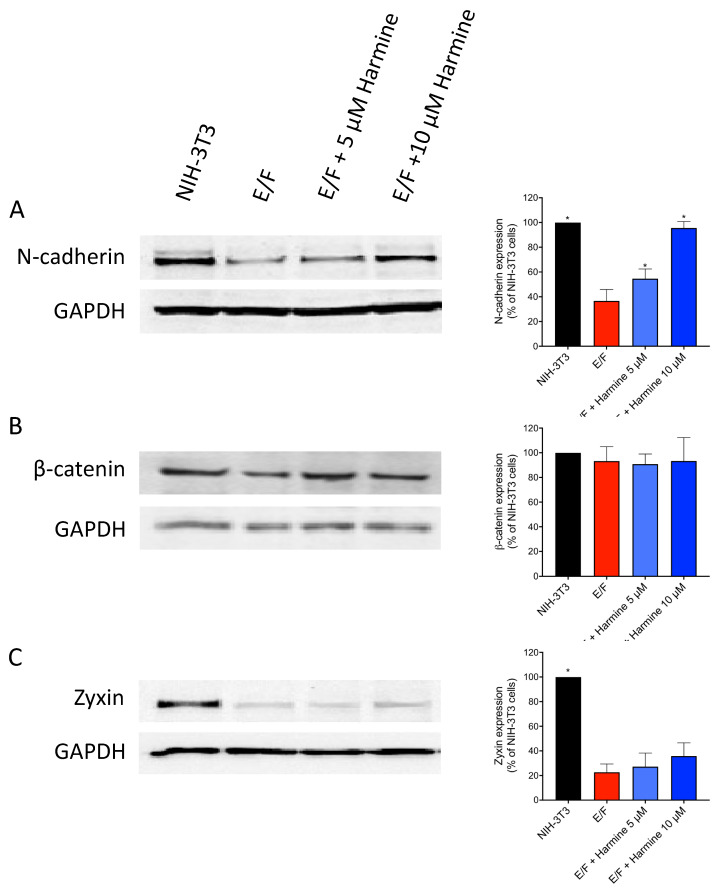
Effect of harmine on the expression of the cell-adhesion-related proteins. NIH-3T3 and E/F cells were cultured in the absence or presence of 5 µM or 10 µM harmine, as indicated. After 72 h of culture, the cells were lysed and proteins analyzed by Western blot using anti-N-cadherin (**A**), anti-β-catenin (**B**), anti-zyxin (**C**) and anti-GAPDH (**A**–**C**) antibodies. The autoradiograms presented are those of a typical experiment. The histograms represent a quantitative analysis of protein expression normalized to NIH-3T3 control cells. The results presented are the means ± SEM for two independent experiments. * *p* < 0.05 versus control untreated E/F cells.

On the other hand, EWS-Fli-1 transformation induces actin cytoskeleton disruption and cell polarization (Figure 2, second column). E/F cells are rather “spindle-shaped”. Zyxin staining in E/F cells is faint and rather diffuse with some puncta at the membrane but no longer associated with cell–matrix or cell–cell contacts (Figure 2A). The junction protein N-cadherin (Figure 2B) and its associated protein β-catenin (Figure 2C) are weakly visible at cell adhesion sites. This loss of membrane localization of these adhesion-related proteins is associated with strong inhibition of the expression level of N-cadherin (around 65% inhibition) and zyxin (around 80% inhibition) in the EWS-Fli1-transformed NIH-3T3 (E/F) cells in comparison to control NIH-3T3 cells (Figure 3). As a result, E/F cells possess very little intercellular contacts and are no longer organized as clusters. Actin remains diffusely distributed within the cytoplasm or accumulates into lamellipodia, which enables cells to have a higher migratory capacity (Figure 2, second column).

Harmine treatment completely changed the morphology of E/F cells most probably as a result of its ability to favor actin polymerization. In fact, harmine-treated tumor cells recovered their actin cytoskeleton with the occurrence of stress fibers (Figure 2, third and fourth columns) and zyxin-rich focal complexes (Figure 2A). The intensified zyxin staining at the focal adhesions and intercellular contacts seems to indicate the relocation and concentration, at these sites, of the zyxin molecules that were already expressed in E/F cells but not a result of an increase in the expression level of this protein (Figure 2A and Figure 3C).

Furthermore, the restoration of intercellular contacts in harmine-treated E/F cells associated with a strong accumulation of N-cadherin (Figure 2B) and β-catenin (Figure 2C) at junction sites enabling cell–cell interconnections as revealed by intense staining. These harmine-induced morphological changes on E/F cells were accompanied by a marked increase in the amount of N-cadherin that reached that of untransformed NIH-3T3 cells (around 96%), as determined by Western blot (Figure 3A). However, as for zyxin, the level of expression of β-catenin does not vary between the control E/F cells and harmine-treated E/F cells (Figure 3B,C), suggesting that harmine simply allows the relocation of these proteins in their functional areas (i.e., plasma membrane, Figure 2A,C).

The approximate percentages of cells exerting a normal-like phenotype (i.e., typical fibroblast shape and a highly organized actin filament network, consisting of numerous, thick stress fibers connected to focal adhesions and intercellular contacts; similar to parental NIH-3T3 cells) were quantified (Figure S4). The results show that while E/F-transformed NIH-3T3 cells have completely lost the NIH-3T3 normal-like phenotype (<2% normal-like cells), their treatment with 5 µM and 10 µM harmine allowed the recovery of this phenotype in around 49.8% and 79.2% of the cells, respectively.

Noteworthy, the treatment of NIH-3T3 untransformed fibroblasts with 10 µM harmine did not seem to affect their actin cytoskeleton organization or cell morphology (Figure S5).

### 3.3. Harmine Increases Cell–cell Adhesion Forces in EWS-Fli1-Transformed (E/F) Cells

**Figure 4:**
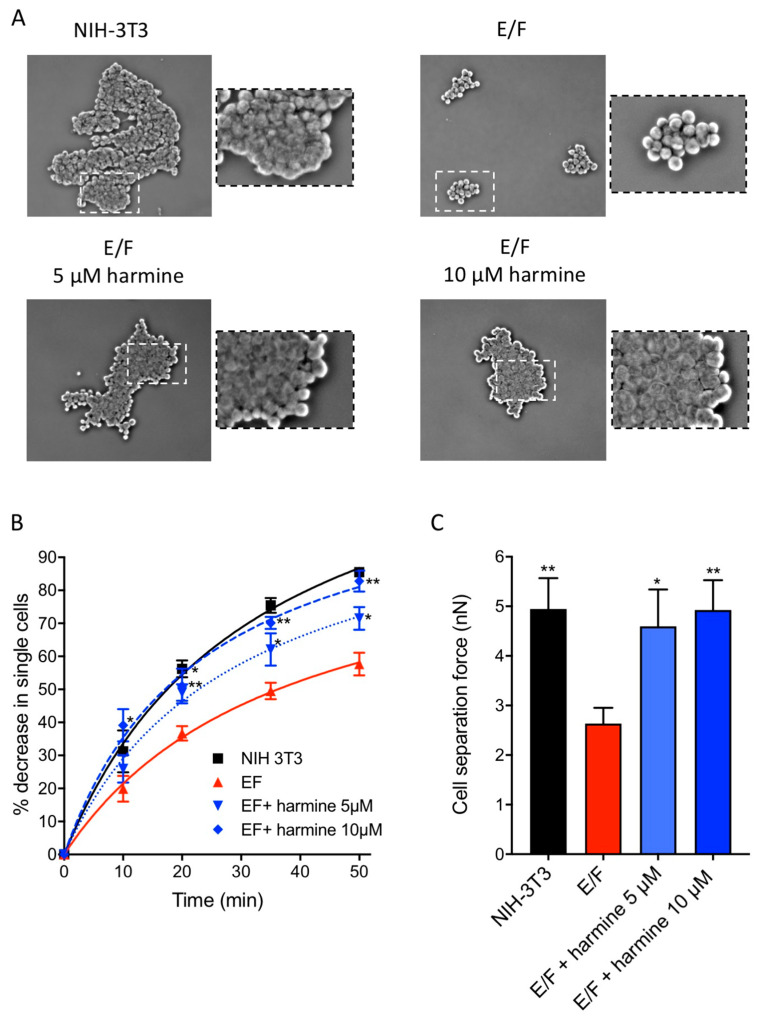
Effect of harmine on the E/F cell aggregation potency and cell separation force. NIH-3T3 and E/F cells were cultured for 48 h in the presence or not of harmine as indicated, then they were gently dissociated. (**A**,**B**) To test the cell aggregation potency, suspensions of isolated cells were shaken during 50 min and cell aggregation was assessed by determining the disappearance of isolated cells over time using a phase-contrast microscopic observation. (**A**) Representative images of cell aggregates after 50 min of shaking from at least three independent experiments (10× objective). (**B**) Graphic representation of the disappearance in isolated cells as a function of time. The results presented are means ± SEM for three independent experiments. * *p* < 0.05; ** *p* < 0.01 versus control untreated E/F cells. (**C**) To test the strength of the intercellular adhesion, two isolated cells are brought into contact for 30 s then the cell doublet separation force is measured using a dual pipette assay. Forces are expressed in nanonewtons. Results are means ± SEM of at least 20 doublet measurements in three independent experiments. * *p* < 0.05; ** *p* < 0.01 versus control untreated E/F cells.

In order to further characterize the effect of harmine on cell–cell adhesion, we carried out two assays that allow the estimation of cell aggregation potency (Figure 4A,B) and the force of intercellular adhesion (Figure 4C).

The cell aggregation assay is a simple test that allows the assessment of the speed at which the single cells aggregate as well as the quality of the intercellular contacts involved. The results presented in Figure 4A,B show a significant difference in behavior between NIH-3T3 and E/F cells. Following shaking of the cell suspensions for 50 min, NIH-3T3 cells form large aggregates whereas E/F cells form small aggregates composed of highly refractive cells indicating the presence of limited contact areas between cells (Figure 4A). At this time, approximately 85% of NIH-3T3 cells are in aggregated form whereas this is the case only for 55% of E/F cells (Figure 4B). E/F cells treated with either 5 µM or 10 µM harmine recover the ability to form aggregates that are similarly large and non-refractive to those observed with NIH-3T3 cells (Figure 4A). Furthermore, harmine treatment dose-dependently accelerates the speed of aggregate formation which, for 10 µM harmine concentration, reaches a level comparable to that of the non-transformed cells (Figure 4B). Thus, harmine enhances the formation of intercellular contacts which seem more numerous.

The intercellular adhesion strength is a function of the number of cadherins expressed on the cell surface and also depends on the organization of the actin cytoskeleton [33]. In order to have the quantitative aspect of the effect of harmine on the strength of the intercellular contacts, we used a dual pipette assay for measuring the forces required to separate two adherent cells maintained in suspension in order to avoid the confounding impact of cell–matrix adhesion [33]. Cell adhesion was initiated rapidly, with cells adhering to each other after only a few seconds of contact. A mean force of about 5 nN (nano Newton) was required to separate adherent NIH-3T3 cells whereas a mean force of about 2.8 nN was sufficient to separate adherent E/F after 30 s of contact (Figure 4C). This result clearly demonstrates the cell–cell adhesion impairment resulting from malignant transformation. Interestingly, the treatment of malignant E/F cells with either 5 µM or 10 µM harmine results in the recovery of cell separation forces with values of 4.8 and 5 nN, respectively, which are very close to those obtained with NIH-3T3 non-transformed cells (Figure 4C). Thus, harmine enhances not only the number of intercellular contacts but also the adhesion strength between the cells. These results also emphasize the direct relations that exist between actin dynamics, cytoskeleton organization and cell–cell adhesion [26,27,34].

### 3.4. Harmine Reduces the Intrinsic Motility of EWS-Fli1-Transformed (E/F) Fibroblasts

**Figure 5:**
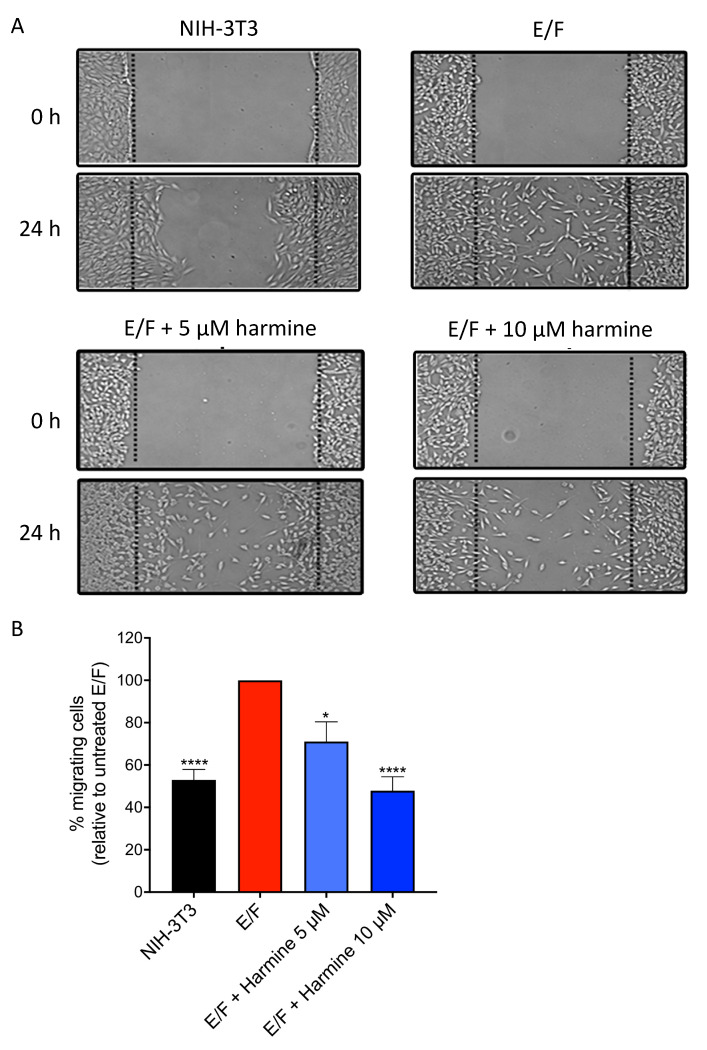
Effect of harmine on E/F cell motility. NIH-3T3 and E/F cells were cultured for 48 h to reach confluency in the presence or absence of harmine, then a wound was made in the monolayer. Cell migration was examined during 24 h by phase-contrast light microscopy. The images of wound repair were taken at 0 h and 24 h after wound. (**A**) Representative images. (**B**) Quantification of the cells that migrated from the wound edges into the wounded area. The results are the means ± SEM for four independent experiments. * *p* < 0.05; **** *p* < 0.0001 versus control untreated E/F cells.

Due to the crucial roles played by cytoskeleton organization and actin polymerization in cell migration which is involved in tumor progression and invasion, it was of interest to determine whether harmine would affect the intrinsic motility of malignant E/F cells. In order to obtain an overall evaluation of their motile properties, we compared the behavior of NIH-3T3, E/F and harmine-treated E/F cells in a wound-healing assay. Cells were grown to confluence (48 h of harmine contact for treated cells) and a wound was created in the cell monolayer with a plastic scraper. Migration of the cells beyond the border of the wound, into the empty space, was monitored 24 h later (Figure 5). The results show that NIH-3T3 cells migrate only slightly into the wound, while maintaining contact with each other. In contrast, E/F cells migrate beyond the border of the wound into its whole area. When treated with harmine (5 µM or 10 µM), less E/F cells (around 30% and 53% decrease, respectively) are found in the central part of the wound compared to untreated E/F control cells. Thus, harmine reduces cell motility of EWS-FLI1-transformed NIH-3T3 fibroblasts.

### 3.5. Harmine Inhibits Anchorage-Independent Growth of EWS-Fli1-Transformed (E/F) Cells at Non-Cytotoxic Concentrations

**Figure 6:**
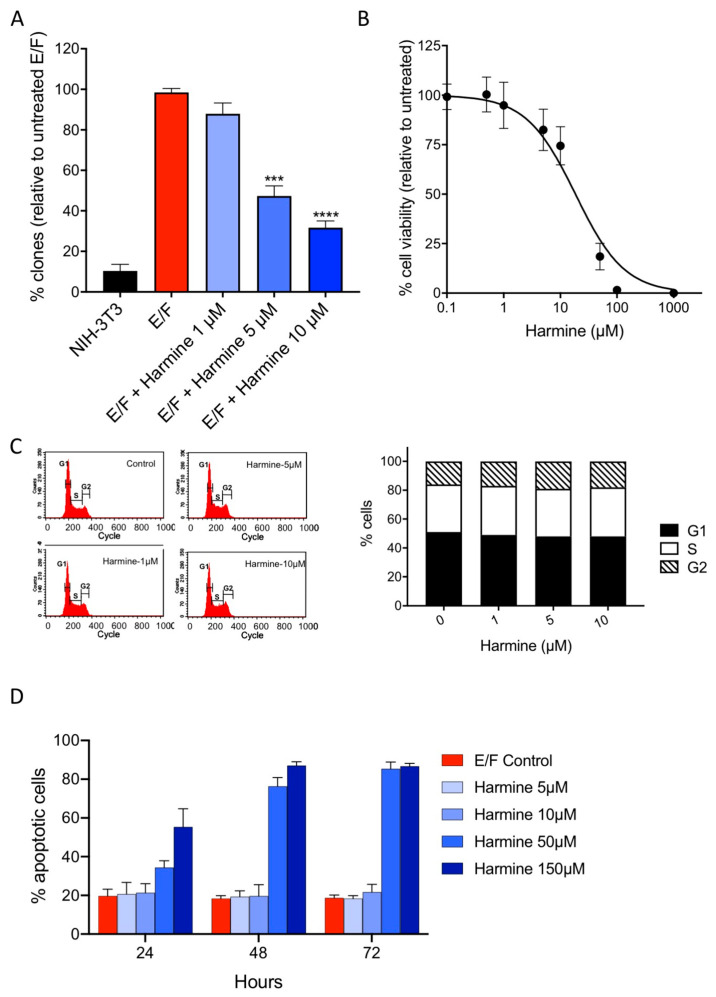
Effect of harmine on E/F cell anchorage-independent growth. (**A**) NIH-3T3 and E/F cells were cultured, in the absence or presence of different concentrations of harmine (as indicated), in methylcellulose semi-solid medium for 3 weeks. Then, the colonies formed were counted. The results are expressed as a percentage of untreated E/F control (means ± SEM) from three independent experiments. (**B**) MTT assay performed on adherent E/F cells cultured in the presence of different concentrations of harmine for 72 h. The results are expressed as a percentage of untreated E/F control (means ± SD) from three independent experiments. (**C**) Flow cytometry analysis of E/F cells cultured during 72 h using standard operating conditions in the presence of increasing concentrations of harmine. Cells were labeled with propidium iodide and the % of cells in either G1/S/G2 phases was estimated. The results presented are from one representative experiment. (**D**) Early detection of apoptosis using Annexin V/propidium iodide staining on E/F cells cultured in the presence of different concentrations of harmine for 72 h. The results are presented as percentages ± SD of Annexin V-positive/propidium iodide-negative cells for three independent experiments.

To further investigate the effect of harmine on the tumor phenotype reversion, we examined whether this molecule affects anchorage-independent growth [35,36,37]. Anchorage-independent growth reflects the ability of malignant cells to survive and proliferate in the absence of signaling from the extracellular matrix mediated principally by integrins and is regulated by the actin cytoskeleton [38]. This feature of oncogenic transformation can be assessed through the evaluation of cell clonogenicity in a semi-solid medium (Figure 6A). Plate analysis showed that cell treatment with increasing concentrations of harmine markedly inhibits the anchorage-independent growth in a dose-dependent manner with an IC_50_ of about 4.5 µM. It should be noticed that harmine cytotoxicity as measured by the MTT test yielded an IC_50_ of 18.5 µM (Figure 6B) and that, consequently, the inhibition of proliferation in the semi-solid medium is most probably not due to a direct cytostatic/cytotoxic effect. This assumption is confirmed, in part, by flow cytometry as shown in Figure 6C which depicts the effect of harmine on the cell cycle of E/F cells cultured in standard adherent conditions. In the absence of a drug, most cells (84%) were in the G_1_ and S phases due to the proliferative state of E/F cells. After 72 h of incubation with 1 µM, 5 µM and 10 µM harmine, no significant variation in the cell cycle phases was observed and, in particular, no sub-G_1_ peak, characteristic of apoptotic cells, was detected. The absence of a harmine-induced cytotoxic effect was further confirmed by the absence of apoptosis, assessed by Annexin V-FITC staining in cells treated for 24–72 h with harmine at concentrations of up to 10 µM, (Figure 6D). In contrast, harmine was cytotoxic at concentrations of 50 µM and above (Figure 6B,D). Taken together, these results indicate that harmine inhibits anchorage-independent growth, a hallmark of the malignant phenotype [35,36,37], and thereby reverts tumor phenotype of EWS-Fli1-transformed NIH-3T3 cells at non-cytotoxic concentrations.

### 3.6. Harmine Inhibits Anchorage-Independent Growth and Reverses Tumor Phenotype of B16-F10 Melanoma Cells at Non-Cytotoxic Concentrations

**Figure 7:**
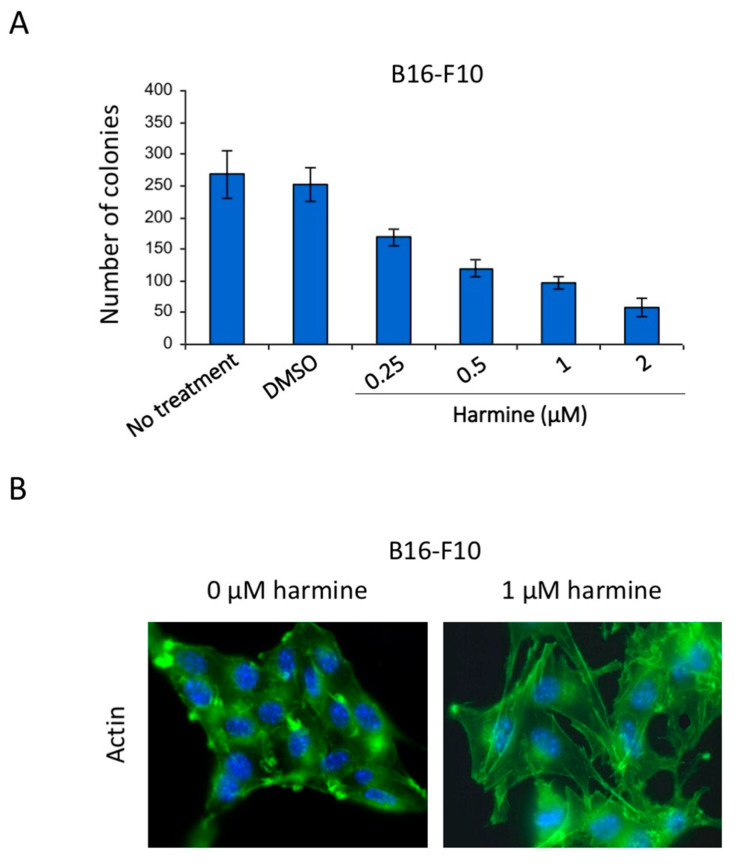
Effect of harmine on B16-F10 melanoma cell anchorage-independent growth, morphology and actin cytoskeleton organization. (**A**) B16-F10 melanoma cells were cultured in the absence or presence of different concentrations of harmine (as indicated), in methylcellulose semi-solid medium for 3 weeks. Then, the colonies formed were counted. The results are expressed as means ± SEM from three independent experiments. (**B**) B16-F10 cells were cultured in the absence of harmine for 2 days then treated or not with 1 µM harmine for 24 h. After treatment, the cells were fixed, permeabilized and co-stained for actin (**Green**). Cell nuclei were stained with DAPI. The cells were analyzed with a fluorescence microscope. Images are representative fields from two independent experiments.

To check whether harmine could, similarly to E/F cells, also reverse the tumor phenotype of other types of cancer, anchorage-independent growth of B16-F10 melanoma cells and their actin cytoskeleton were analyzed in the presence of non-cytotoxic concentrations of harmine (Figure 7 and Figure S6). The results show that in contrast to the other β-carbolines of *Peganum harmala* (harmol, harmane, harmaline, harmalol, Figure S6), harmine strongly inhibits anchorage-independent growth of B16-F10 melanoma cells with an IC50 of 0.5 µM (Figure 7A). Furthermore, in the absence of harmine, B16-F10 cells display a round shape with the unstructured actin cytoskeleton (Figure 7B, left image). Treatment of these tumorigenic cells with 1 µM harmine for 24 h induces significant morphological changes from round to fibroblastoid shape accompanied by the actin cytoskeleton reorganization into stress fibers characteristic of non-tumorigenic cells (Figure 7B, right image). However, since B16-F10 cells already grow in vitro as clusters with high intercellular adherence, harmine treatment could not visibly further affect their cell–cell contacts. Nonetheless, the harmine-induced loss of anchorage-independent growth and morphological change characteristic of non-tumorigenic cells indicate that this molecule potently induces tumor phenotype reversion of melanoma cells at non-cytotoxic concentrations.

## 4. Discussion

A number of direct observations show that the actin cytoskeleton is disrupted in numerous cancer cells and experimental tumor cell models [1,5,6,12,18]. Alteration of actin assembly is an active contributor to the malignant transformation rather than simply being a by-product of cellular transformation. NIH-3T3 cells expressing the oncoprotein EWS-Fli1 (E/F cells) exhibit such characteristics. In this model, malignant transformation is accompanied by dramatic changes in morphology, disorganized actin cytoskeleton, a higher rate of motility, enhanced anchorage-independent growth and tumorigenicity in nude mice. We have shown previously [26] that disruption of the actin cytoskeleton correlates with a reduced expression of zyxin in NIH-3T3 fibroblasts transformed by EWS-Fli1. Zyxin, acts as a molecular scaffold to facilitate the assembly of multiprotein complexes that promote actin polymerization [39]. When re-expressed in the E/F-transformed cells, zyxin specifically suppresses EWS-Fli1-induced malignant phenotype. This tumor suppressor role of zyxin was confirmed in a human Ewing tumor-derived cell line (SKNMC) which also produces low constitutive levels of zyxin [26]. Thus, it is possible that a drug mimicking the effects of zyxin could suppress the malignant phenotype in the absence of a direct cytotoxic effect. In order to isolate such molecules, we have developed an in vitro assay that allows the assessment of actin polymerization by fluorescence anisotropy in cellular extracts [28]. Using this screening assay linked to a secondary screening assay that evaluates cell–cell adhesion recovery, we were able to identify harmine, that increases actin polymerization in vitro and recovers actin filament network organization and cell–cell adhesion in transformed cells, as a potential drug candidate.

Although drugs facilitating actin polymerization have been identified previously (jasplakinolide, phalloïdine, dolastatine) [12], their clinical development was terminated at an early stage, due to their high toxicity. In most cases, these molecules bind to actin filaments resulting in actin condensation leading to cytotoxicity. In contrast, we found, by direct pyrene-actin polymerization assay, that harmine does not interact directly with actin filaments. This is further supported by the low cytotoxicity of harmine and the absence of actin condensation in harmine-treated cells, in contrast to the effect of jasplakinolide as indicated above. Importantly, when harmine is assessed at non-cytotoxic doses, the morphology of E/F cells approaches the fibroblast morphology of parental NIH-3T3 cells and their cytoskeleton is restructured. In addition to the recovery of the fibroblastic cellular morphology, actin remodeling is accompanied by the loss of anchorage-independent growth and the inhibition of cell migration. Consistently to the E/F model, non-cytotoxic of harmine induces actin cytoskeleton reorganization and inhibits anchorage-independent growth in B16-F10 melanoma cells.

The process of anchorage-independent growth in vitro is a key aspect of the tumor phenotype, particularly with respect to the metastatic potential and is widely used as a marker for in vitro transformation [35,36,37]. In fact, it reflects the ability of malignant cells to survive and proliferate in the absence of signaling from cell–matrix adhesion receptors, principally the integrins and is regulated by the actin cytoskeleton [38]. This property is essential to enable cancer cells to detach and escape the primary tissue, survive in the circulatory and/or lymphatic systems and colonize new sites in the body to form metastasis. In contrast, most normal cells (including fibroblasts and epithelial cells) depend on the survival signals coming from the cell–matrix adhesion receptors and die in the absence of attachment to a proper substratum [36]. Consistently, several studies comparing the in vitro and in vivo behavior of cancer cells and using microarray gene expression analysis identified the anchorage-independent growth assay as a valuable in vitro assay that correlates with in vivo behavior and metastatic potential of cancer cells and; therefore the in vitro anchorage-independent growth ability is widely accepted as a surrogate phenotype of malignancy [35,36,37]. Thus, our results clearly confirm that a pharmacological approach targeting the restructuration of the actin cytoskeleton could also abrogate the malignant phenotype of cancer cells as in the E/F model system. However, harmine induces the reversion of the tumor phenotype of EWS-Fli1-transformed cells without recovering the expression level of zyxin, suggesting that this molecule acts through a mechanism that is different from that observed in the case of re-expression of zyxin in these cells [26]. The mechanism by which harmine modulates actin dynamics and cytoskeleton organization remains to be elucidated.

Previous studies have shown that, at high concentrations (above 15–20 uM), harmine exerts significant anti-proliferative and cytotoxic (pro-apoptotic) activities in different types of cancer, consistently with our observations. In these studies, harmine acted with IC50s ranging from 50 to 160 uM [40,41,42]. At high concentrations, harmine was found to act by several mechanisms, described in different cell types and experimental conditions. These mechanisms include inhibition of the expression levels of the TAZ transcriptional co-activator [40], the epithelial–mesenchymal transcription factor TWIST [42] and STAT3 transcription factor [41], inhibition of the phosphorylation of ERK and Akt [40,43], as well as inhibition of the anti-apoptotic Bcl2 protein and induction of the pro-apoptotic Bax protein [40]. However, those mechanisms were not observed at lower harmine concentrations. On the other hand, harmine has been previously identified as a potent inhibitor of DIRK1A, a dual-specificity kinase that possesses both serine/threonine and tyrosine kinase activities [44]. Harmine can directly inhibit DIRK1A activity in hippocampal neurons and inhibit neurite formation at low concentrations (1.6 uM) [44]. Although the function of DIRK1A was not identified in this study, other studies describe the role of this kinase in actin cytoskeleton regulation. In fact, Park et al. have shown that Dyrk1A negatively regulates the actin cytoskeleton through threonine phosphorylation of N-WASP (Neural Wiskott–Aldrich syndrome protein). Dyrk1A directly phosphorylates the GTPase-binding domain (GBD) of N-WASP at three sites (Thr196, Thr202 and Thr259), which promotes the intramolecular interaction of the GBD with verprolin, cofilin and acidic (VCA) domains of N-WASP, and subsequently inhibits Arp2/3-complex-mediated actin polymerization [45]. Furthermore, DIRK1A can phosphorylate dynamin 1, thereby preventing its interaction with various SH3 containing partner proteins such as endophilin, intersectin and pascin [46]. All these proteins are known to affect cytoskeletal organization, and some of them, such as endophilin A1, have been shown to promote actin polymerization [47]. However, whether the role of harmine in the regulation of actin polymerization and the induction of the tumor phenotype reversion in E/F cells could be mediated by its effect on DIRK1A still needs to be tested. Thus, further studies will be required to identify the specific molecular target of harmine responsible for actin remodeling in tumor cells.

## 5. Conclusions

In conclusion, we identified the β-carboline, harmine, as an inducer of the reversion of the malignant phenotype in transformed cells by a process involving the modulation of actin dynamics. Our study suggests that harmine is a potential anti-tumor agent acting principally through a non-cytotoxic process, which could spare normal cells and circumvent apoptotic defects in cancer cells.

## Supplementary Materials

The following are available online at https://www.mdpi.com/2073-4409/9/5/1168/s1, Figure S1: Effect of *Peganum harmala seed* methanolic extract on actin polymerization. Figure S2. Effect of harmine and japlakinolide on actin polymerization in E/F cell extracts. Figure S3: Dose–response analysis of jasplakinolide and harmine on actin polymerization in the presence of E/F cell extracts by fluorescence anisotropy (A) or on pure actin by pyrene-actin polymerization assay (B). Figure S4: Effect of harmine on the reversion of E/F-transformed cell morphology into a normal-like phenotype. Figure S5. Effect of harmine on NIH-3T3 actin cytoskeleton and cell morphology. Figure S6: Effect of harmine and other β-carbolines of *Peganum harmala* on B16-F10 cloning efficiency in semi-solid medium.
