# Supplementary material for: The β-carboline Harmine Induces Actin Dynamic Remodeling and Abrogates the Malignant Phenotype in Tumorigenic Cells
Source: Cells. 2020 May 8;9(5):1168. doi: 10.3390/cells9051168 (PMC7290983; doi:10.3390/cells9051168)
Supplement: Supplementary file 1 [file cells-09-01168-s001.pdf]

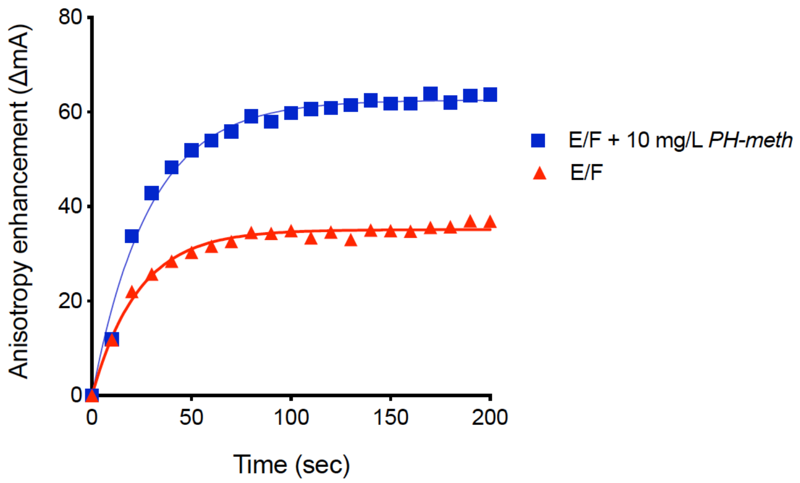

Figure S1. Effect of *Peganum harmala* seed methanolic extract on actin polymerization. Kinetics of F-actin linear polymerization as measured in E/F cell extracts using the fluorescence anisotropy assay. When indicated, 10 mg/L methanolic extract of *Peganum harmala* seeds (*PH*-meth) is added at time 0 to the polymerization buffer, actin-Alexa488 and E/F extracts. Actin polymerization was measured at different incubation times using fluorescence anisotropy assay.

A

	ΔmA_{eq} (AU)	T_{eq} (sec)	K_{eq} (sec^{-1})
E/F	43	99.27	0.038
E/F + 10 µM harmine	59	51	0.08
E/F + 10 µM jasplakinolide	62.1	30	0.137

B

	$\Delta mA_{1/2}$ (AU)	$T_{1/2}$ (sec)	$K_{1/2}$ (sec^{-1})
E/F	21.5	20.5	0.034
E/F + 10 µM harmine	29.5	6.76	0.1
E/F + 10 µM jasplakinolide	31.05	5.3	0.13

Figure S2. Effect of harmine and jasplakinolide on actin polymerization in E/F cell extracts**.** F-actin linear polymerization was measured, using the fluorescence anisotropy assay, in E/F cell extracts treated with vehicle, 10 µM harmine or 10 µM jasplakinolide as in Figure 1C. The anisotropy value (ΔmA), rate constant of actin elongation (K) and time to reach this value (T) were calculated at the steady state corresponding to the reaction equilibrium (eq) and at the half of equilibrium reaction (1/2) using the equation: Y = Ymax.[1-exp(-K.X)] where Y= ΔmA and T = time.

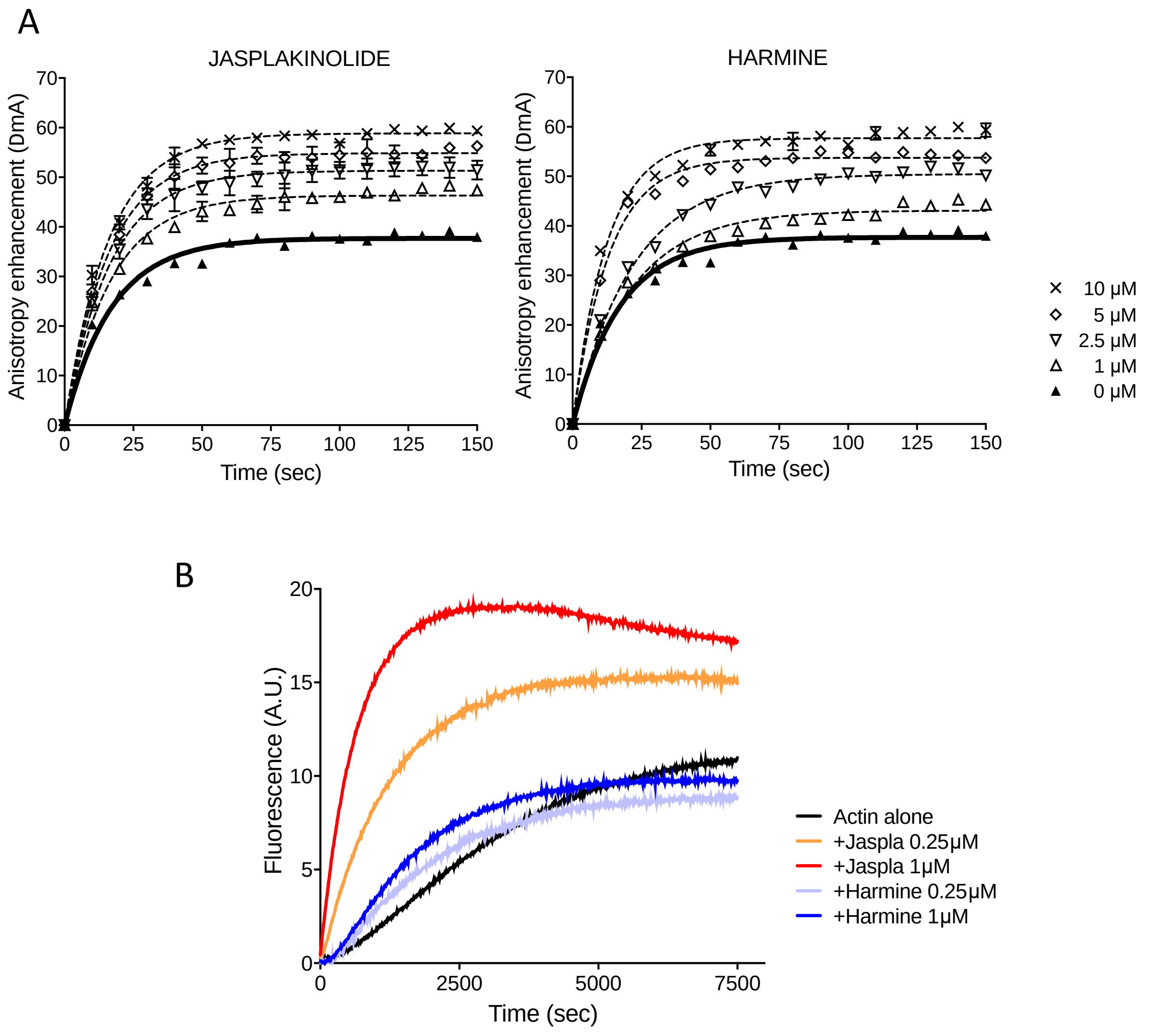

Figure S3. Dose-response analysis of jasplakinolide and harmine on actin polymerization in the presence of E/F cell extracts by fluorescence anisotropy (**A**) or on pure actin by pyrene-actin polymerization assay (**B**). (**A**) Effect of jasplakinolide and harmine on actin polymerization in the presence of cellular extracts. Jasplakinolide (left graph) or harmine (right graph) are added at the indicated concentrations to the polymerization buffer, actin-Alexa488 and E/F extracts. Actin polymerization was measured at different incubation times using fluorescence anisotropy assay. Bars represent mean ± SD from two independent experiments. (**B**) Effect of jasplakinolide and harmine on pure actin polymerization. Jasplakinolide (left graph) or harmine (right graph) are added at the indicated concentrations to 10% pyrene-actin mixed with the polymerization buffer. The polymerization of the fluorescent G-actin is followed up by the measurement, at every second, of the increase in fluorescence using a fluorescent spectrophotometer.

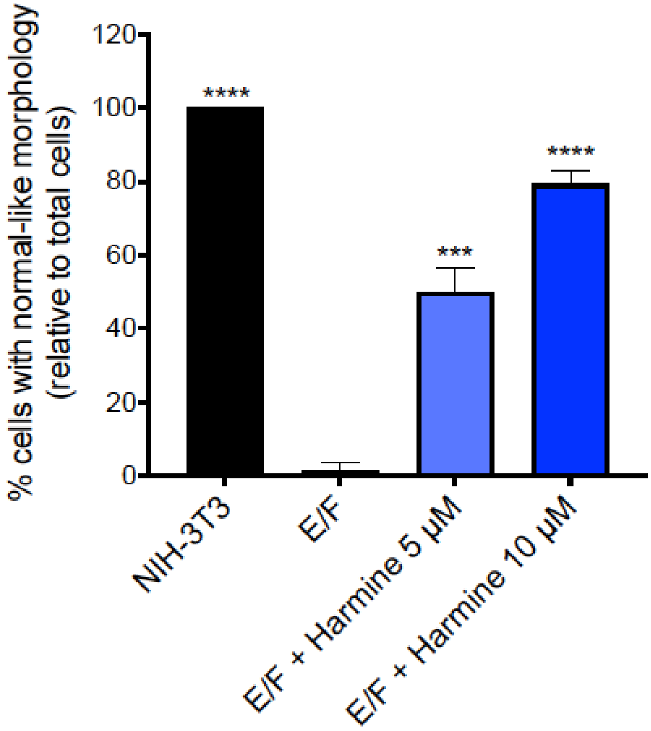

Figure S4. Effect of harmine on the reversion of E/F-transformed cell morphology into a normal-like phenotype. NIH-3T3 and E/F cells were cultured in the absence or presence of 5 µM or 10 µM harmine. After 72 hours of culture, the cells were fixed, permeabilized and co-stained for actin with one of the following adhesion-related proteins; zyxin, N-cadherin or β-catenin. Cell nuclei was stained with DAPI. Then, the cells were analyzed with a fluorescence microscope. The images are presented in figure 2 of the manuscript. The cells presenting a normal-like phenotype (typical fibroblast shape and a highly organized actin filament network, consisting of numerous, thick stress fibers connected to focal adhesions and intercellular contacts; similar to parental NIH-3T3 cells) were counted for each condition in different representative fields from three independent experiments. The results are the means ± SD for three independent experiments. ***$p < 0.001$; ****$p < 0.0001$ versus control untreated E/F cells.

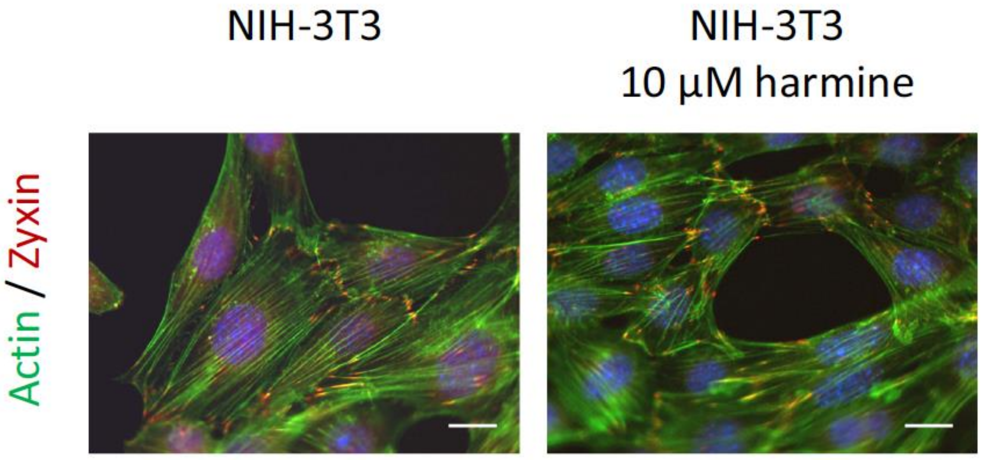

Figure S5. Effect of harmine on NIH-3T3 actin cytoskeleton and cell morphology. NIH-3T3 cells were cultured in the absence or presence of 10 µM harmine. After 72 hours of culture, the cells were fixed, permeabilized and co-stained for actin (green) and zyxin (red). Cell nuclei was stained with DAPI

(blue). Then, the cells were analyzed with a fluorescence microscope. Images are representative fields from one experiment. Scale bars: 10 μm.

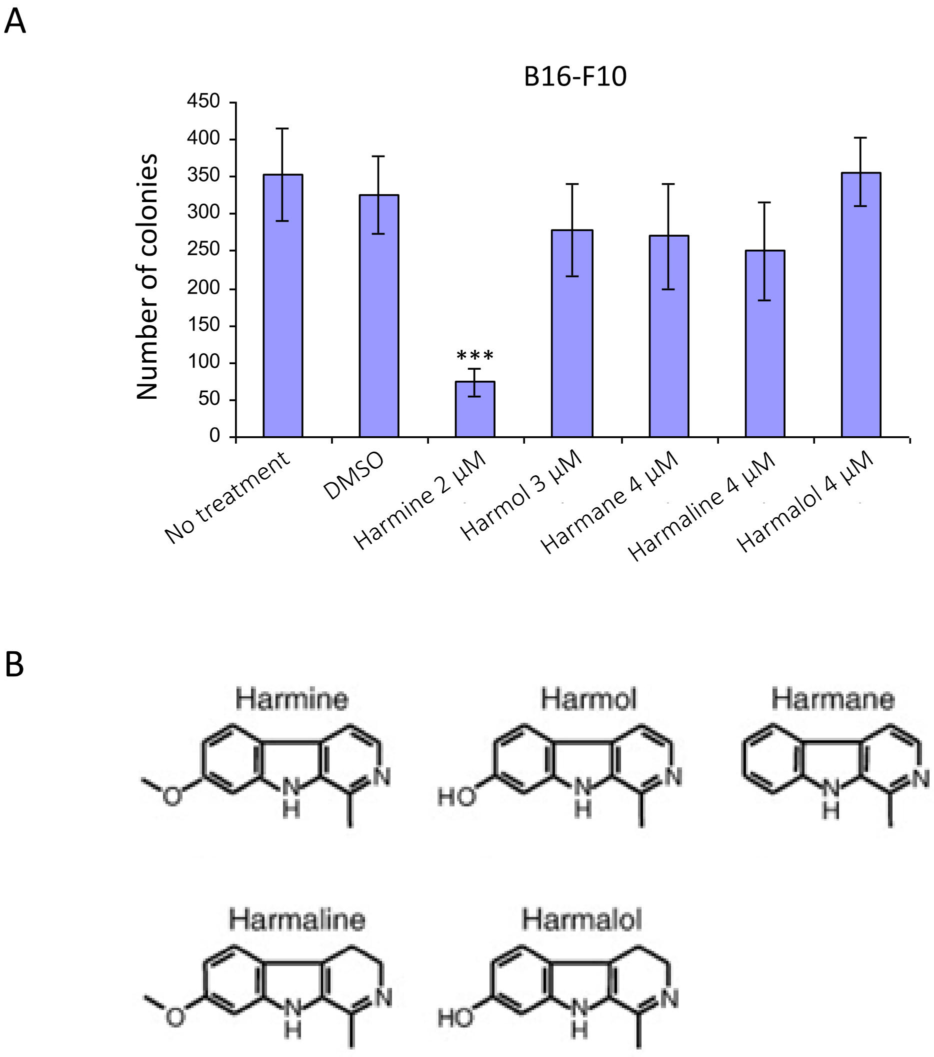

Figure S6. Effect of harmine and other β-carbolines of *Peganum harmala* on B16-F10 cloning efficiency in semi-solid medium. (**A**) B16-F10 melanoma cells were cultured, in the absence or presence of vehicle, harmine or other β-carbolines at the indicated concentrations, in methylcellulose semi-solid medium for 3 weeks. Then, the colonies formed were counted. The results are means ± SD from four independent experiments. ***$p < 0.001$ versus DMSO-treated control. (**B**) Chemical structures of harmine, harmol, harmane, harmaline and harmalol.
